# Expression of cytochrome P450 mRNAs in Type II alveolar cells from subjects with chronic obstructive pulmonary disease

**DOI:** 10.1002/prp2.405

**Published:** 2018-05-21

**Authors:** Satoshi Kamata, Naoya Fujino, Mitsuhiro Yamada, Ken Grime, Satoshi Suzuki, Chiharu Ota, Yukiko Tando, Yoshinori Okada, Akira Sakurada, Masafumi Noda, Yasushi Matsuda, Hisatoshi Sugiura, Masakazu Ichinose

**Affiliations:** ^1^ Department of Thoracic Surgery Institute of Development, Aging and Cancer Tohoku University Sendai Japan; ^2^ Department of Respiratory Medicine Tohoku University Graduate School of Medicine Sendai Japan; ^3^ Respiratory Inflammation & Autoimmunity IMED Biotech Unit AstraZeneca Gothenburg Sweden; ^4^ Department of Thoracic Surgery Japanese Red Cross Ishinomaki Hospital Ishinomaki Japan; ^5^ Department of Advanced Preventive Medicine for Infectious Disease Tohoku University Graduate School of Medicine Sendai Japan

**Keywords:** alveolar epithelial type II cells, chronic obstructive pulmonary disease, cytochrome P450 enzymes, lung

## Abstract

Inhaled drugs are critical for the treatment of inflammatory airway diseases such as chronic obstructive pulmonary disease (COPD). To develop better therapeutics for pulmonary disease it is of potential importance to understand molecular mechanisms of local biotransformation in the lung. Alveolar epithelial type II (ATII) cells have a key role in homeostasis in the lung, but little is known about expression patterns of genes encoding cytochrome P450 (CYP) enzymes in ATII cells. In addition, alteration of CYP gene expression has not been fully defined in COPD. We previously established a method to purify ATII cells from the adult human lung using fluorescence‐activated cell sorting. By employing this technique we determined gene expression patterns of 14 CYP enzymes in ATII cells from nonsmokers (n = 4) and smokers (n = 4), both having normal pulmonary function. Although most CYP genes are highly expressed in primary hepatocytes, we found that *CYP1B1* mRNA expression was 7.2‐fold higher in ATII compared to hepatocytes (*P* = .0275). Additionally we noted a 3.0‐fold upregulation of *CYP2C19* and 50% reduction in *CYP2J2* mRNA expressions in ATII cells isolated from patients with COPD (n = 3) compared to smokers without COPD (n = 4). These data, for the first time, detail a comprehensive set of genes encoding CYP enzymes in human ATII cells and highlights differentially expressed CYP mRNAs of patients with COPD. Such understanding may have important implications for the development of novel inhaled drugs.

**Abbreviations**
%FEV1: percent predicted forced expiratory volume in 1 s
ATII cell: an alveolar epithelial type II cell
COPD: chronic obstructive pulmonary disease
CYP: cytochrome P450
EETs: epoxyeicosatrienoic acids
FACS: fluorescence‐activated cell sorting
FEV1: force expiratory volume in 1 s
FOXA3: forkhead box protein A3
FVC: forced vital capacity
PAH: polycyclic aromatic hydrocarbon
PAHs: polycyclic aromatic hydrocarbons

## 1 INTRODUCTION

Inhaled drugs such as long‐acting beta‐agonists, long‐acting antimuscarinic antagonists, and inhaled corticosteroids are used as therapeutic options for patients with chronic obstructive pulmonary disease (COPD)^1^ which is a leading cause of morbidity, mortality, and health‐care costs worldwide.^2^ In addition to the current treatments, recent advances indicate that next‐generation inhaled drugs including kinase inhibitors have been developed for inflammatory airway diseases.^3^ This is because therapeutic agents that are directly administered to the lungs have at least 2 substantial merits: (i) the rapid onset of action by direct delivery to the airways and (ii) the local high concentration to minimize the systemic adverse effects. Susceptibility to drug metabolism in the target organ has the possibility of reducing effective drug concentration and producing metabolites that give rise to patient safety risk or have pharmacological activity. Additionally, a novel candidate drug may beneficially inhibit an enzyme responsible for producing potentially toxic metabolites of an inhaled molecule that may be present in, for example, tobacco smoke. Thus development of inhaled drugs ideally requires in‐depth knowledge of drug biotransformation in the lung.

Expression and functional activity of drug‐metabolizing enzymes including cytochrome P450 (CYP) enzymes can be altered in diseases of the liver which is the vital organ for metabolisms of orally administered therapeutics.^4^ The human pulmonary tissue also expresses a wide range of CYP enzymes to metabolize a great variety of inhaled xenobiotics and toxicants, but the expression pattern of lung CYPs substantially differ from metabolisms in the liver.^5^, ^6^, ^7^ A recent study using a comprehensive expression analysis for genes encoding membrane transporters and phase I and II drug‐metabolizing enzymes indicated that these gene expressions were not significantly altered in lung tissues from patients with COPD compared to healthy subjects.^8^ However, given previous reports indicating different expression patterns of drug‐metabolizing enzymes in cell populations such as alveolar epithelial cells, pulmonary vascular endothelial cells, and airway macrophages,^9^, ^10^ cell‐type‐specific analyses are required to precisely define differences in CYP gene expression in lung tissues between healthy subjects and patients with pulmonary diseases. In the alveolar region alveolar epithelial type I (ATI) and type II (ATII) cells, together with alveolar macrophages constitute the major cellular population. While ATI and ATII cells make up approximately 90 and 3% of the alveolar surface area, respectively, ATII cells have been designated defenders of the alveolus due to their highly differentiated function including role in surfactant synthesis, importance in the repair process after lung injury.^11^, ^12^

Among component cells in lung alveolar walls, an alveolar epithelial type II (ATII) cell has a critical role in homeostasis of the alveolar epithelium which is directly exposed to inhaled xenobiotics or compounds.^11^, ^12^ Although previous reports using immunohistochemistry demonstrated the presence of CYP1A1, CYP1B1, and CYP3A5 proteins in ATII cells in human lungs,^9^, ^10^ no studies have comprehensively clarified expression patterns of CYPs in human ATII cells. Furthermore, to our knowledge, alteration in the CYP gene expression in human ATII cells has not been investigated in inflammatory airway diseases, such as COPD.

In this study, we aimed to determine expression patterns of 14 genes encoding CYP enzymes in human ATII cells using a fluorescence‐activated cell sorting (FACS)‐based method for purifying ATII cells from human lung tissues.^13^ In addition, we sought to determine CYP enzymes that were differentially expressed in ATII cells of patients with COPD.

## 2 MATERIALS AND METHODS

### 2.1 Patients and human lung tissue preparation

The Ethics Committees at Tohoku University School of Medicine and at Japanese Red Cross Ishinomaki Hospital approved this study. The experiments conformed to the principles set out in the WMA Declaration of Helsinki. All subjects gave their informed consent. Patients’ characteristics are shown in Table 1. Human lung tissues without any pathological abnormalities including tumors, overt inflammation and fibrosis were obtained from patients who underwent lung resection for primary lung cancer at the Department of Thoracic Surgery at Tohoku University Hospital or at Japanese Red Cross Ishinomaki Hospital. Resected lung tissues were immediately immersed in a tissue preserve solution (Stem Survive; Kurabo, Osaka, Japan). We used 1‐2 g of lung tissues and isolated cells within 6 h after surgery.

**Table 1:** Patients’ characteristics

Patient	Age	Gender	Smoking (Pack‐years)	FEV1/FVC	%FEV1	GOLD stage	Treatment for COPD	Lobe resected
Nonsmoker1	70	F	0	78.0	122.9	N.A.	N.A.	RU
Nonsmoker2	81	F	0	70.2	98.9	N.A.	N.A.	LU
Nonsmoker3	78	F	0	78.9	104.2	N.A.	N.A.	RL
Nonsmoker4	69	F	0	84.0	94.2	N.A.	N.A.	RL
Smoker1	76	M	61.5	91.8	117.0	N.A.	N.A.	LL
Smoker2	81	M	60	82.0	92.7	N.A.	N.A.	LU
Smoker3	44	M	1	83.6	106.5	N.A.	N.A.	RU
Smoker4	62	M	60	82.0	94.0	N.A.	N.A.	RU
COPD1	70	M	127.5	51.3	78.0	A	LAMA	RL
COPD2	79	M	11.5	61.2	77.8	A	LABA	LU
COPD3	51	M	30	68.1	73.3	A	LAMA	RU

COPD, chronic obstructive pulmonary disease; FEV1, force expiratory volume in 1 s; FVC, forced vital capacity; %FEV1, percent predicted forced expiratory volume in 1 s; GOLD, Global Initiative for Chronic Obstructive Pulmonary Disease; LAMA, long‐acting muscarinic antagonist; LABA, long‐acting β2 agonist; N.A., not applicable; RU, right upper lobe; LU, left upper lobe; RL right lower lobe; LL, left lower lobe.

### 2.2 Isolation of alveolar epithelial type II cells from human lungs

ATII cells were isolated from human lung tissues as previously described.^13^ Briefly, single‐cell suspensions obtained from lung tissues were incubated with the following antibodies: Alexa Fluor 700‐mouse anti‐human CD45 antibody (clone HI30; Biolegend); phycoerythrin‐mouse anti‐human EpCAM antibody (clone 1B7; eBioscience); Alexa Fluor 647‐rat anti‐human T1α antibody (clone NC‐08; Biolegend); fluorescein isothiocyanate‐mouse anti‐human VE‐cadherin antibody (clone 55‐7H1; BD Pharmingen). 7‐amino actinomycin D (eBiosciences) was used to eliminate dead cells. A live/single cell gated CD45‐negative/EpCAM‐high/T1α‐low/VE‐cadherin‐negative cell subset was sorted as an ATII cell population by FACS Aria II cell Sorter and FACS Diva version 6.1 (BD Biosciences). This routinely resulted in more than 90% of prosurfactant protein‐C‐positive ATII cells. Sorted cells were put into Buffer RLT Plus (Qiagen) and stored at −80°C until RNA extraction and quantitative reverse transcription‐polymerase chain reaction (qPCR).

### 2.3 Preparation of human hepatocytes

Primary human hepatocytes were obtained from Kaly‐Cell (Plobsheim, France). Three single donors (67‐year‐old male, 64‐year‐old female and 42‐year‐old female) were used for experiments. Total RNA was extracted from freshly thawed cells for a gene expression analysis.

### 2.4 RNA extraction and quantitative reverse transcription‐polymerase chain reaction

Total RNA of human ATII cells and human hepatocytes were isolated using All Prep Micro Kit (Qiagen). The quantity and quality were assessed by NanoDrop‐2000 (ThermoFisher Scientific). Total RNA was reverse‐transcribed using QuantiTect Reverse Transcription Kit (Qiagen) according to the manufacturer's instruction with genomic DNA elimination. The following Taqman probe sets were used (Applied Biosystems): *CYP1A1*, Hs01054797_g1; *CYP1A2*, Hs00167927_m1; *CYP1B1*, Hs02382916_s1; *CYP2A6*, Hs00868409_s1; *CYP2B6*, Hs04183483_g1; *CYP2C8*, Hs02383390_s1; *CYP2C9*, Hs02383631_s1; *CYP2C19*, Hs00426380_m1; *CYP2D6*, Hs00164385_m1; *CYP2E1*, Hs00559367_m1; *CYP2J2*, Hs00356035_m1; *CYP2U1*, Hs00766273_m1; *CYP3A4*, Hs00604506_m1; *CYP3A5*, Hs04273722_m1; *GAPDH*, Hs99999905_m1. *GAPDH* was used as an endogenous control. Real‐time PCR was conducted using the StepOne Plus Real‐Time PCR system (Applied Biosystems) according to the manufacturer's instruction. The relative expression levels of the specific mRNAs were calculated using the ΔΔC_t_ method.

### 2.5 Statistics

All data are presented as the mean ± SEM. Statistical analyses were conducted using GraphPad Prism (GraphPad Software). To compare 2 datasets, 2‐tailed unpaired *t*‐test was applied. Correlation was evaluated by Spearman's rank‐order correlation. *P* < .05 was considered significant.

## 3 RESULTS AND DISCUSSION

We firstly sought to determine expression patterns of genes encoding 14 CYP enzymes in human ATII cells by comparing to those in human hepatocytes that are major cell‐types metabolizing orally or systemically administered drugs. We isolated ATII cells from non‐COPD patients (nonsmokers, n = 4; smokers, n = 4) whose tissues were pathologically confirmed not to have overt inflammation or fibrotic changes. Among 14 CYP genes tested, *CYP1A2* and *CYP3A4* mRNAs were not detected in the isolated ATII cells (data not shown). These 2 enzymes are major CYP isoforms in human liver.^14^ The data presented here agrees previous studies showing no or very low expressions of *CYP1A2* and *CYP3A4* mRNAs in human lung tissue.^8^, ^15^

**Figure 1:**
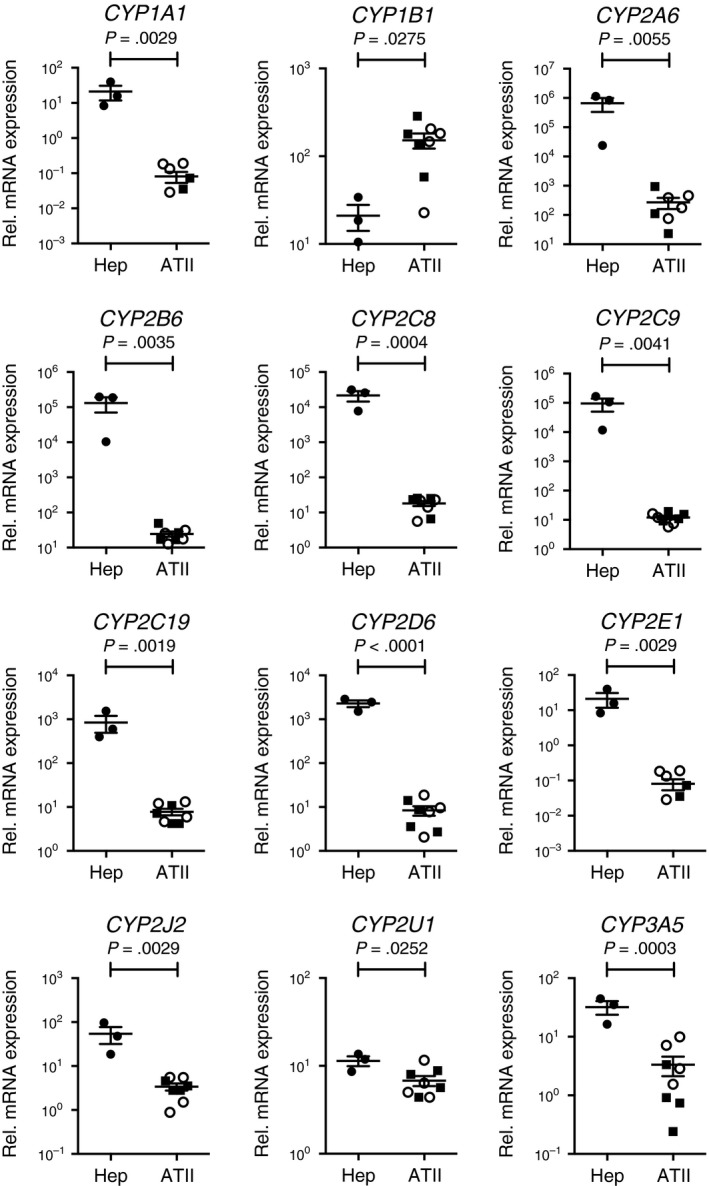
mRNA expression patterns for 12 CYP enzymes between human primary hepatocytes (Hep, n = 3) and isolated alveolar epithelial type II cells (ATII, n = 8). n is the number of individuals. Each dot represents an individual and bars indicate the mean ± SEM. Two‐tailed unpaired *t*‐test was used for statistics. Open circles, never‐smokers; Closed squares, smokers without COPD

In the remaining 12 CYP genes only *CYP1B1* mRNA significantly increased in ATII cells compared to hepatocytes (Figure 1). CYP1B1 metabolically activates polycyclic aromatic hydrocarbons (PAHs) such as Benzo[*a*]pyrene contained in tobacco smoke to ultimately generate mutagenic and carcinogenic compounds.^16^ Considering recent studies using rat ATII cells indicated that proinflammatory cytokines such as tumor necrosis factor‐α augmented genotoxic effects of PAHs through CYP1B1,^17^, ^18^ our results give extra weight and importance to the role of such a mechanism in the development of lung carcinoma under proinflammatory conditions.

Although other CYP genes were found to be at approximately 10‐fold (3A5, 2J2), 100‐fold (2C19, 1A1, 2E1, 2D6) or 1000‐fold (2C8, 2A6, 2B6, 2C9) higher levels in hepatocytes than in ATII cells (Figure 1), there was only 2‐fold difference in the expression level of CYP2U1 between the 2 cell‐types. This is of particular interest since although the expression of CYP2U1 has previously been shown to be at a lower level in the lung,^19^ the enzyme is postulated to be selectively expressed in the brain and thymus of human organs 20 and to metabolize arachidonic acids to ultimately generate 19‐ or 20‐Hydroxyeicosatetraenoic acid.^21^ Since arachidonic acid‐derived eicosanoids such as prostaglandins and leukotrienes regulate immune cellular functions in inflammatory airway diseases,^22^ our data suggest that additional focus on the role of CYP2U1 in arachidonic acid metabolism on alveolar epithelium in proinflammatory settings may be warranted.

**Figure 2:**
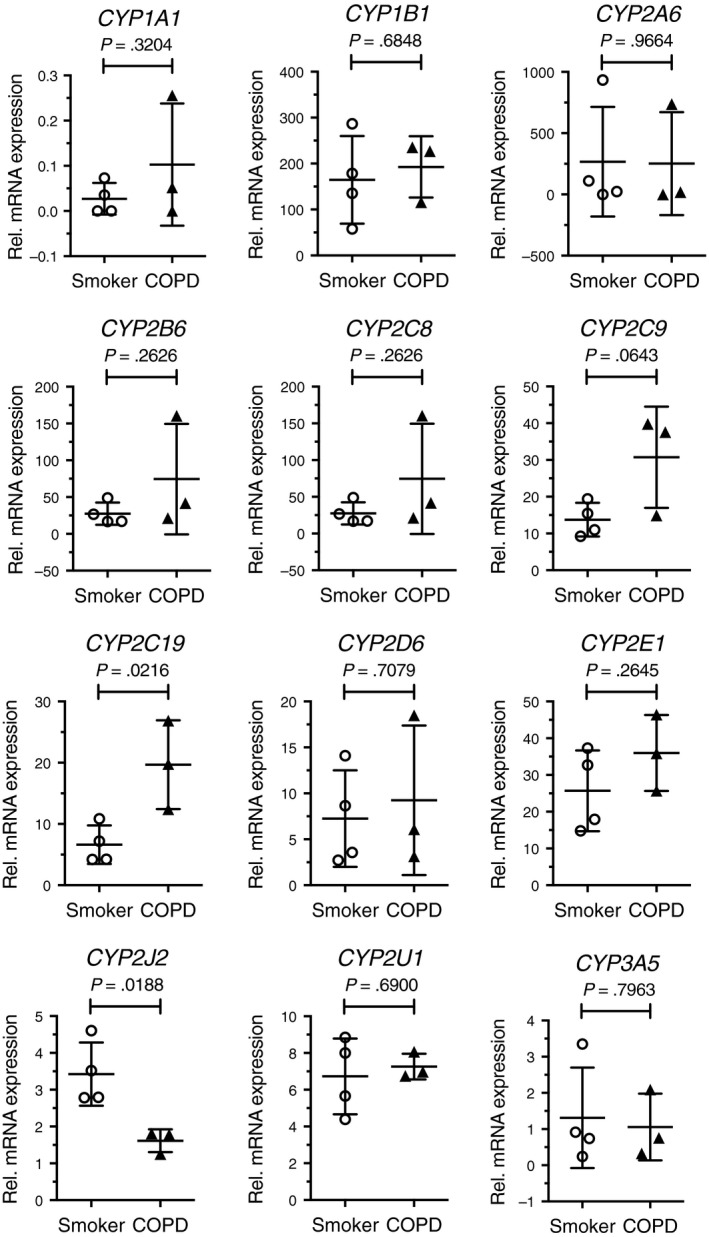
Comparison of mRNA expression of 12 CYP enzymes in ATII cells between smokers without COPD (n = 4) and patients with COPD (n = 3). n indicated the number of individuals. Each dot represents an individual and bars indicate the mean ± SD. Two‐tailed unpaired *t*‐test was used for statistics

A comparison was made for the mRNA expression of the 12 CYP genes in ATII cells from healthy smokers and patients with COPD. Although the sample number was relatively small, *CYP2C19* mRNA in ATII cells of COPD patients showed 3.0‐fold higher levels than that in ATII cells from smokers (Figure 2). In contrast, *CYP2J2* mRNA in COPD subjects was half the level of that in smokers (Figure 2). These differences were not identified when whole‐lung tissues from smokers and COPD patients were used for analysis.^8^

CYP2C19 is a major phase 1 enzyme in the liver and is involved in metabolism of a wide range of drugs such as proton pomp inhibitors and anticancer agents.^23^ Although genetic polymorphisms are translated to the poor metabolism of these drugs in some individuals, recent studies have supported the idea that factors regulating *CYP2C19* transcription are also important for its pharmacokinetics.^24^ One such transcription factor responsible for transactivation of *CYP2C19* is forkhead box protein A3 or FOXA3.^25^ The reason for the higher expression of CYP2C19 in ATII cells from COPD patients (Figure 2) may be due to the upregulation of FOXA3 in inflamed airway epithelium of these patients.^26^ This data provides a rationale for the investigation of FOXA3 in the transregulation of *CYP2C19* gene expression in COPD.

CYP2J2 is expressed in extrahepatic tissues, especially in the heart, but also in the lung^19^ and is known to metabolize endogenous arachidonic acids for generating epoxyeicosatrienoic acids (EETs)^27^ as well as exogenous diverse therapeutics such as antihistamine drugs.^28^ Recent studies using mouse models indicate that CYP2J2‐mediated EETs reduce excessive inflammation in the lung and liver.^29^, ^30^ Thus the reduced *CYP2J2* expression in COPD ATII cells may contribute to proinflammatory conditions in disease.

In summary, we clarified expression patterns of genes encoding cytochrome P450 enzymes in alveolar epithelial type II cells (ATII) in human lung and found higher expression of *CYP1B1* mRNA in ATII cells than in primary hepatocytes. Moreover using the cell‐type‐specific approach we for the first time demonstrated that *CYP2C19* and *CYP2J2* mRNA expressions were altered in ATII cells in COPD. These CYP enzymes are known to be able to metabolize kinase inhibitors.^31^ Taken together with promising roles of kinase inhibitors for the treatment of COPD,^32^ our results lead to a new insight into the altered expression of CYP enzymes in the alveolar epithelium in inflammatory airway diseases.

## ACKNOWLEDGEMENTS

We thank the Biomedical Research Core of Tohoku University Graduate School of Medicine and Biomedical Research Unit of Tohoku University Hospital for technical supports.

## DISCLOSURE

None declared.

## AUTHOR CONTRIBUTION

Fujino, Yamada, and Grime participated in research design; Kamata, Ota, Tando, Suzuki, Sakurada, Noda, and Matsuda conducted the experiments; Kamata, Fujino, Grime, and Yamada performed data analysis; Kamata, Fujino, Yamada, Grime, Okada, Sugiura, and Ichinose wrote or contributed to the writing of the manuscript.
